# A Real-World Longitudinal Case-Study Implementing Digital Screening and Treatment for Distress in Inflammatory Bowel Disease: The COMPASS-IBD Patient Journey

**DOI:** 10.1093/ibd/izaf259

**Published:** 2026-01-13

**Authors:** Annie S K Jones, Natasha Seaton, Sophie Harding, Alexa Duff, Joanna Hudson, Abigail Wroe, Sam Norton, Harinder Singh, Jemima Onih, Rona Moss-Morris

**Affiliations:** Health Psychology Section, Department of Psychology, Institute of Psychiatry, Psychology and Neuroscience, King’s College London, London, United Kingdom; Department of Psychological Medicine, Faculty of Medical and Health Sciences, University of Auckland, Auckland, New Zealand; Health Psychology Section, Department of Psychology, Institute of Psychiatry, Psychology and Neuroscience, King’s College London, London, United Kingdom; Health Psychology Section, Department of Psychology, Institute of Psychiatry, Psychology and Neuroscience, King’s College London, London, United Kingdom; Health Psychology Section, Department of Psychology, Institute of Psychiatry, Psychology and Neuroscience, King’s College London, London, United Kingdom; Health Psychology Section, Department of Psychology, Institute of Psychiatry, Psychology and Neuroscience, King’s College London, London, United Kingdom; Health Psychology Section, Department of Psychology, Institute of Psychiatry, Psychology and Neuroscience, King’s College London, London, United Kingdom; Department of Inflammation Biology, Faculty of Life Sciences and Medicine, King’s College London, London, United Kingdom; Lead Patient and Public Involvement Representative, London, UK; IMPARTS (Integrating Mental and Physical healthcare: Research, Training and Services), King’s Health Partners, London, United Kingdom; Health Psychology Section, Department of Psychology, Institute of Psychiatry, Psychology and Neuroscience, King’s College London, London, United Kingdom

**Keywords:** distress, integrated care, IBD

## Abstract

**Background:**

Comorbid psychological distress (anxiety and depression) in inflammatory bowel disease (IBD) is common and associated with poorer outcomes and increased healthcare burden. Scalable and accessible integrated care is needed. This study examined the feasibility of implementing routine digital mental health screening and digital cognitive-behavioral therapy (COMPASS-IBD) for psychological distress in a large IBD service.

**Methods:**

During implementation, distress was identified by screening or IBD clinician referral. Further triage determined eligibility to receive COMPASS-IBD with trainee therapist support (12 weeks). Pre- and post-intervention outcomes examined reach, acceptability, implementation, and potential effectiveness of the new pathway.

**Results:**

Screening was completed by 827 patients (from November 2022 to September 2023), with 196 patients meeting clinical cutoffs and referred for IBD psychology triage. An additional 82 patients were directly referred via IBD clinicians. Of 91 eligible patients, 65 (71.4%) were enrolled into COMPASS-IBD. Distress significantly reduced post-intervention (Patient Health Questionnaire Anxiety and Depression Scale = −6.203; 95% confidence interval, −8.76 to −3.64; *P < *.001; Cohen’s *d = −*0.553). Symptoms of anxiety, depression, and IBD-related quality of life significantly improved, but IBD symptomatology did not. Full adherence (≥5 online and ≥3 therapist sessions) to COMPASS-IBD was completed by 32.3% of patients. After initially increasing, the IBD psychology waitlist decreased in wait time (30.8%) and number (63.4%) by the end of study implementation. Patients were accepting of the new treatment pathway.

**Conclusions:**

Routine mental health screening and COMPASS-IBD were successfully implemented in an outpatient IBD service, but support from trainee psychologists and the research team was required. This new integrated pathway can identify and treat psychological distress in IBD with minimal service resource.

Key Messages **What is already known?**

Psychological distress is common and undertreated in people living with inflammatory bowel disease (IBD), which leads to poorer health outcomes and greater healthcare burden.

**What is new here?**

We developed and implemented a new integrated digital pathway to identify and treat psychological distress in an outpatient IBD service, which was found to be feasible to deliver and effective in improving distress.

**How can this study help patient care?**

This new integrated pathway could provide scalable support to ensure the psychological well-being needs of people living with IBD are met in routine care, which could ultimately improve patient morbidity and healthcare costs.

## Introduction

Inflammatory bowel disease (IBD), largely comprising ulcerative colitis (UC) and Crohn’s disease (CD), is a chronic autoimmune condition characterized by episodic inflammation and remission within the gastrointestinal tract. Common IBD symptoms include pain, fatigue, and urgency. The current prevalence of IBD in the United Kingdom is approximately 0.81%,^1^ with a significant portion of patients experiencing recurrent inflammatory “flares” which lead to morbidity, hospitalization, and disability. IBD therefore incurs substantial annual treatment costs (∼£3000 and ∼£6000 per patient for UC and CD, respectively), which escalate with the severity of the disease.^2^ Mitigating the burden of IBD on patients and the healthcare system is therefore a critical healthcare objective.

Psychological distress, including depression and anxiety, commonly co-occurs with IBD, with prevalence rates as high as 39% and 58% respectively, during periods of flare.^3^ Recent UK data from 10 000 patients living with IBD showed that 89% reported experiencing difficulties coping with their IBD in the past year.^4^ However, 60% of these patients had not been asked about their mental health during their IBD care.^4^ Co-occurring distress results in poorer patient outcomes, including increased inflammation and hospitalization.^5^ Additionally, co-occurring mental health disorders in IBD can elevate healthcare costs by up to 55.5%.^6^ A recent meta-analysis found that psychological interventions to improve distress can reduce inflammation levels in IBD,^7^ suggesting that treating comorbid distress could also improve clinical outcomes.

This demonstrates the need for an efficient treatment pathway within IBD care, which identifies patients experiencing comorbid distress and offers scalable psychological treatments. Previous studies suggest that integrated psychological screening and one-to-one support in routine IBD care is acceptable and effective at treating anxiety and depression.^8^^,^^9^ However, a 2021 survey found that only 2% of IBD services in the United Kingdom adhere to national IBD Standards for psychological provision; a 0.5 whole-time equivalent psychologist per 250 000 people in the catchment area,^4^ making it clear that most patients have no in-service access to face-to-face therapy.^10^

Digital interventions can overcome barriers of traditional face-to-face therapies^10^ by providing on-demand, remote, scalable, and accessible psychological support.^11^ One such intervention, COMPASS, a Web-based cognitive behavioral therapy (CBT) program, delivers support tailored to the challenges of living with a long-term physical health condition (LTC), based on the transdiagnostic model of adjustment in LTCs.^12^ A recent randomized controlled trial (RCT) demonstrated the efficacy of COMPASS (alongside guided therapist support) in treating psychological distress related to living with an LTC, compared with standard LTC charity support alone (standardized mean difference = 0.71). Psychological distress was measured as a composite of anxiety and depression symptoms, and almost half the cohort in this RCT were living with IBD.^13^ These IBD participants provided feedback that further tailoring of COMPASS to the unique challenges of living with IBD would enhance the helpfulness of the program.^14^

The current mixed-methods study aimed to assess the feasibility of implementing a novel integrated digital pathway for identifying and treating psychological distress as part of routine care within a large National Health Service (NHS) gastroenterology clinic in the United Kingdom. The new pathway included (1) routine digital mental health screening to identify psychological distress, (2) further assessment and triage following a positive screen to determine appropriate treatment pathways, and (3) a new version of COMPASS tailored for patients with IBD (COMPASS-IBD) delivered as a treatment for those who met eligibility criteria. The study objectives were to (1) examine the implementation, reach, and acceptability of the new treatment pathway; and (2) establish the potential effectiveness of the treatment pathway for both IBD patients and the service at 12 weeks from the start of intervention.

## Methods

### Study design and participants

This real-world longitudinal study included a nested qualitative study and exploratory analysis of secondary biological outcomes, the methods and results of which will be reported separately.^15^ The study took place within a London-based NHS gastroenterology center, caring for approximately 5000 IBD patients. This service employed 2 clinical psychologists covering 1 whole-time equivalent. Most therapy delivered by the service was in a group format. Patients could be referred to IBD psychology by any healthcare professional (HCP) within the gastroenterology service. The new integrated pathway (see Appendix 1) included routine digital mental health screening, triage (including screening for inclusion in the COMPASS-IBD study), and the COMPASS-IBD therapy. Triage was conducted by 5 clinical psychology doctoral trainees who were completing 6-month training placements in the IBD service at the time of the study (at no cost to the service).

The protocol is published^16^ and registered on ClinicalTrials.gov (NCT05330299). The study received ethical approval from the Health Research Authority and from North West–Greater Manchester East Research Ethics Committee (22/NW/0224). The study was conducted between December 2, 2022, and July 31, 2024.

#### New integrated screening and triage

Digital mental health screening (IMPARTS; https://imparts.org/about/) was implemented into 5 outpatient clinics within the IBD service. Patients attending these clinics received a Web link via text to complete the digital mental health screening with each appointment reminder; however, this was sent no more than once every 3 months. Any patient who screened positive for mild-to-severe distress (see cutoffs in Measures) was eligible for triage to determine treatment pathways (including COMPASS-IBD). As per IMPARTS standard screening procedures, patients only progressed to complete the full Patient Health Questionnaire (8-item version) (PHQ-8)^17^ and Generalized Anxiety Disorder Scale (GAD-7)^18^ if they had a score of 2 or more on the initial items of the PHQ-2 or GAD-2.^19^^,^^20^ Patients could also continue to be referred to the IBD psychology team for support as per usual care throughout the study period.

The triage process is detailed in our published study protocol paper,^16^ with outlines of these processes provided in Appendices 1 and 2. In summary, triage was conducted by clinical psychology doctoral trainees via telephone. Patients with mild-to-severe distress, with no active suicidal ideation, and who related their distress at least in part to the challenges of living with IBD were eligible for referral to the COMPASS-IBD program. COMPASS-IBD study inclusion criteria were (1) being 18 years of age or older, (2) having a diagnosis of IBD (clinical diagnosis from a gastroenterologist, extracted from the hospital clinical records), (3) being proficient in English, (4) having access to a computer/device to complete intervention, (5) consenting to take part in research procedures, and (6) having evidence of (at least) mild symptoms of distress (based on clinical cutoffs) and/or awaiting treatment from the IBD psychology service. Originally, cutoffs were scoring >10 on either the PHQ-8^17^ or GAD-7.^18^ However, as the flow into the study was lower than expected, this was lowered to >5 on either scale during study implementation (May 16, 2023) after consulting the clinical psychologist within the service. This lower threshold allowed patients with milder symptomology to be triaged for eligibility to receive psychological support (including for COMPASS-IBD). During triage, patients were also assessed for PTSD symptomology (see Appendix 2), given that COMPASS is not designed to treat PTSD, which can be prevalent in IBD populations.^21^ Therefore, where PTSD symptomology was present (see Appendix 2 for cutoffs), the trainee and patient discussed the patient’s primary treatment concern to determine whether COMPASS was appropriate. If the patient’s primary treatment concern was PTSD, the trainee discussed more appropriate referral pathways with the patient. Exclusion criteria for the COMPASS-IBD study were (1) substance dependency, cognitive impairment, or a severe mental health condition; (2) active suicidal risk (recent serious suicidal intent and/or planning); (3) receiving (or on a waitlist to receive) psychological treatment outside of the service within the next 6 months (patients could complete COMPASS-IBD while remaining on the IBD psychology service waitlist); and (4) mental health presentation unrelated to IBD. A risk protocol, in line with standard hospital service procedures, was followed if patients displayed active suicidal risk during triage (ie, that they may act on their suicidal thoughts). In such circumstances, clinicians were urgently notified, crisis or emergency services were engaged when required (eg, following disclosure of overdose or severe self-harm), safeguarding concerns were addressed, suicide safety plans were developed where appropriate, and all actions were documented. If a patient voiced suicidal thoughts but denied acting on these, the healthcare team and General Practitioner (GP) were still notified and safeguarding and suicide safety plans were agreed.

#### COMPASS-IBD sample size calculation

An estimated sample size target of 108 was based on recruitment feasibility, rather than on statistical power (given the exploratory nature of the study), focusing on estimating rates of reach, adoption, implementation, and acceptability. This was calculated by estimating that the prevalence of clinical depression and/or anxiety in the sample would be 30% (n = 1500 of the ∼5000-patient caseload in the service). Of people identified as having depression and/or anxiety symptoms, we anticipated approaching 40% (n = 600), with 60% of those approached being eligible for COMPASS-IBD (n = 360) and 30% consenting to take part (n = 108). A sample size of 108 allows for the estimation of 95% binomial exact confidence intervals (CIs) with a maximum width of ±10%.

### COMPASS-IBD procedure

Eligible patients who expressed interest in COMPASS-IBD were contacted by the research team, who completed remote study recruitment procedures (via the online survey platform Qualtrics; https://www.qualtrics.com). After providing consent, participants were emailed the online (via Qualtrics) preintervention questionnaire (capturing demographic and clinical characteristics as well as psychosocial outcomes of interest, see Appendix 3) and subsequently enrolled into the COMPASS-IBD program by the research team. Participants were contacted by their COMPASS-IBD guide (one of the clinical psychology doctoral trainees), who introduced them to the program and booked in their support sessions across the 12 weeks of the intervention. Participants also completed study questionnaires at 12 weeks (post-intervention; measuring changes in clinical outcomes and psychosocial outcomes).

COMPASS guides (clinical psychology trainees) were provided COMPASS-specific training, including triage process training, study protocol training, risk training, specific COMPASS program technology training, and a training manual and specific COMPASS guide scenario training, and were supervised by the clinical psychologist within the gastroenterology service (a routine part of clinical training) as well as an external qualified clinical psychologist specializing in COMPASS.

#### COMPASS-IBD intervention

The current study investigated a new version of COMPASS tailored for patients with IBD. The team worked alongside an IBD patient advisory group to develop COMPASS-IBD, which includes personal stories and relevant scenarios from people living with IBD and additional content relevant to IBD patient experiences, including managing IBD medication, thoughts and behaviors related to food, and the impact of IBD on personal and healthcare relationships. The details of this process will be reported elsewhere.^22^

While COMPASS-IBD was tailored to address IBD-specific concerns, the fundamental components, delivery method, and CBT techniques of the original COMPASS intervention remained unchanged. COMPASS is an interactive, tailored 12-week Web-based CBT program, which people complete in their own time at home. Patients are offered six 30-minute sessions with a COMPASS-trained guide (conducted via video or phone call, and supported by in-session messaging). The 11 online modules feature psychoeducation, patient narratives (discussing the real-life challenges of living with IBD), goal setting, and interactive exercises, all of which utilize evidence-based CBT techniques. These elements are organized across various topics, including managing the uncertainties associated with living with a long-term condition, managing negative thoughts and symptoms, and achieving a routine (among others). Patients can choose which sessions they focus on and are not expected to complete all 11 sessions. More detailed information regarding the COMPASS program, therapist role, and session structure has been published elsewhere.^13^

Upon completion of COMPASS-IBD, the guide’s clinical judgment was used to determine whether patients should be discharged from the psychology pathway or transferred to or remain on the IBD psychology waitlist for further one-to-one therapy. Patients requiring additional support beyond the scope of the IBD psychology service were referred to external services, as per standard care.

### Outcomes

Measurement operationalization of each construct, including all measures and outcomes, is summarized in Appendix 3. Further details of the measures are provided in the published protocol.^16^

#### Primary implementation outcomes

**Reach*:***

*What is the percentage of and what are the demographic and clinical characteristics of IBD patients who:*

- Complete mental health screening?
- Report clinical levels of distress at screening?
- Complete triage?
- Meet COMPASS-IBD inclusion criteria?
- Consent to participate in the COMPASS-IBD intervention?

**Implementation:**

- What is the level of adherence to COMPASS-IBD? (Composite adherence was defined as attending ≥3 therapist appointments and completing ≥5 online sessions, as specified in the COMPASS RCT.^13^ The minimum optimal dose has not yet been calculated, but this threshold was decided by experienced clinical and health psychologists who developed COMPASS.
- What are the percentages of COMPASS-IBD nonengagers (enrolled but never use COMPASS-IBD) and dropouts (disengage after initial use)?
- How many patients require digital onboarding for COMPASS-IBD?

**Effectiveness:**

*Patient level:*

- What are the changes from pre- and post-intervention in self-reported clinical outcomes? The primary outcome was psychological distress, measured using the PHQ Anxiety and Depression Scale (PHQ-ADS),^23^ which combines the PHQ-9 (9-item version),^24^ and GAD-7^18^ into a 16-item measure of distress with scores ranging from 0 to 48. Higher scores indicate greater distress, and a decrease of ≥4 is considered a minimum clinically important ­difference.^23^ Secondary outcomes were symptoms of anxiety (GAD-7) and depression (PHQ-9) measured separately (higher scores on both measures reflect greater depressive [range 0-24] and anxious [range 0-19] symptomology, respectively), IBD-related quality of life (UK IBD Questionnaire)^25^ and IBD symptomatology (Patient Global Impression Scales of Severity and Improvement (12-week only).^26^
- Do the putative therapeutic mechanism outcomes change pre-post COMPASS-IBD? These include:
  - Illness self-management, measured by the Self-Efficacy of Managing Chronic Disease Scale,^27^ a 6-item measure with higher scores indicating greater confidence in managing symptoms and illness-related challenges.
  - Illness perceptions, measured by the Brief Illness Perception Questionnaire,^28^ an 8-item measure whereby items are summed, with higher scores indicating a more threatening perception of illness.
  - Cognitive and behavioral responses to illness, measured by the Cognitive and Behavioral Responses Questionnaire.^29^ This is an 18-item measure with higher scores indicating more unhelpful responses.
  - Acceptance of IBD, measured by the Acceptance of Chronic Health Conditions scale,^30^ a 10-item measure with higher totals indicating a greater acceptance of illness.

*Service level:*

- How many patients are stepped up to (or removed from) the IBD psychology waitlist after completing COMPASS-IBD?
- What is the change in the IBD psychology waitlist pre-post COMPASS-IBD implementation? Note that this outcome was incorrectly labeled as “adoption” in the protocol paper.^16^

**Acceptability:**

- How satisfied are patients with the new treatment pathway (measured at 12 weeks only using the theoretical framework of acceptability (TFA)^31^?
- What is the extent to which HCPs consider the new treatment pathway part of routine care? What resources are required to sustain the new pathway? The second acceptability outcome (see Outcomes) will be reported in a ­separate paper.^32^

#### Progression criteria

Composite progression criteria of red, amber, and green (RAG) ratings for key outcomes informed decisions regarding whether to recommend proceeding to a full-scale hybrid type II trial (see Table 1). Participation rate, adherence to COMPASS (composite measure), and potential effectiveness (mean change on PHQ-ADS) were the target outcomes within the reach, implementation, and effectiveness objectives, respectively.

**Table 1.** Summary of reach objective outcomes.

**Reach** ** outcome**	Data
**Completed digital mental health screening**	827 (24.9)^a^
**Reported clinical levels of distress at screening**	
** Anxiety (GAD-7)**	158 (19.1)
** Depression (PHQ-8)**	156 (18.9)
** Distress (PHQ-ADS)**	181 (21.9)
**Completed triage (of those referred n = 278)**	139 (50)
**Met COMPASS-IBD inclusion criteria (of those who completed triage)**	91 (65.5)
**Consented to complete the COMPASS-IBD intervention**	71 (78.0)

a Denominator is the estimated number of contacts made to patients over the screening period by the DrDoctor system, which distributed the text with the screening link (n = 3324; further details in results section).

### Statistical analysis

Implementation outcomes were analyzed descriptively with frequencies and percentages for categorical variables, and ­continuous variables were reported as mean ± SD or median (interquartile range) as appropriate. Any estimates of rates (eg, screening rate) or mean differences (eg, mean change from baseline) relating to the key outcomes were reported with 95% CIs to represent uncertainty in the estimates. Differences in patient-level outcomes between demographic and clinical characteristics at baseline were examined using linear or logistic regression (depending on the nature of the outcome). The models included age, sex, and race/ethnicity as covariates, with clinical variables added sequentially to separate models. Internal consistency for all patient level outcomes was assessed by ­Cronbach’s alpha (Appendix 3).

Change from baseline for potential effectiveness outcomes and potential mechanism of action variables were assessed using mixed-effects linear regression models, with a random intercept to account for repeated assessments within individuals. Assessment point (preintervention or 12 weeks) was entered into models as a dummy-coded covariate to estimate change from baseline, along with adjustment for age, sex, race/ethnicity, and self-reported diagnosis of anxiety and/or depression. For comparability with RCTs, effect sizes were reported as Cohen’s *d* with small sample correction based on the adjusted mean change from baseline in the mixed-effects models. The denominator here is the pooled standard deviation, treating the baseline assessment as the control group and the post-treatment assessment as the intervention group. We also report standardized response means, in which the denominator is the standard deviation of the change scores, to provide information about the responsiveness of outcomes. An additional, per-protocol sensitivity analysis was conducted for the potential effectiveness outcomes, only including participants who met the composite adherence criteria. Moderator analyses were conducted for the primary outcome with the intention-to-treat sample to examine treatment effect heterogeneity across age, sex, race/ethnicity, education level, IBD subtype and self-reported diagnosis of anxiety and depression. All analyses were undertaken using Stata Version 18 (StataCorp).

## Results

### Reach

**Figure 1.**
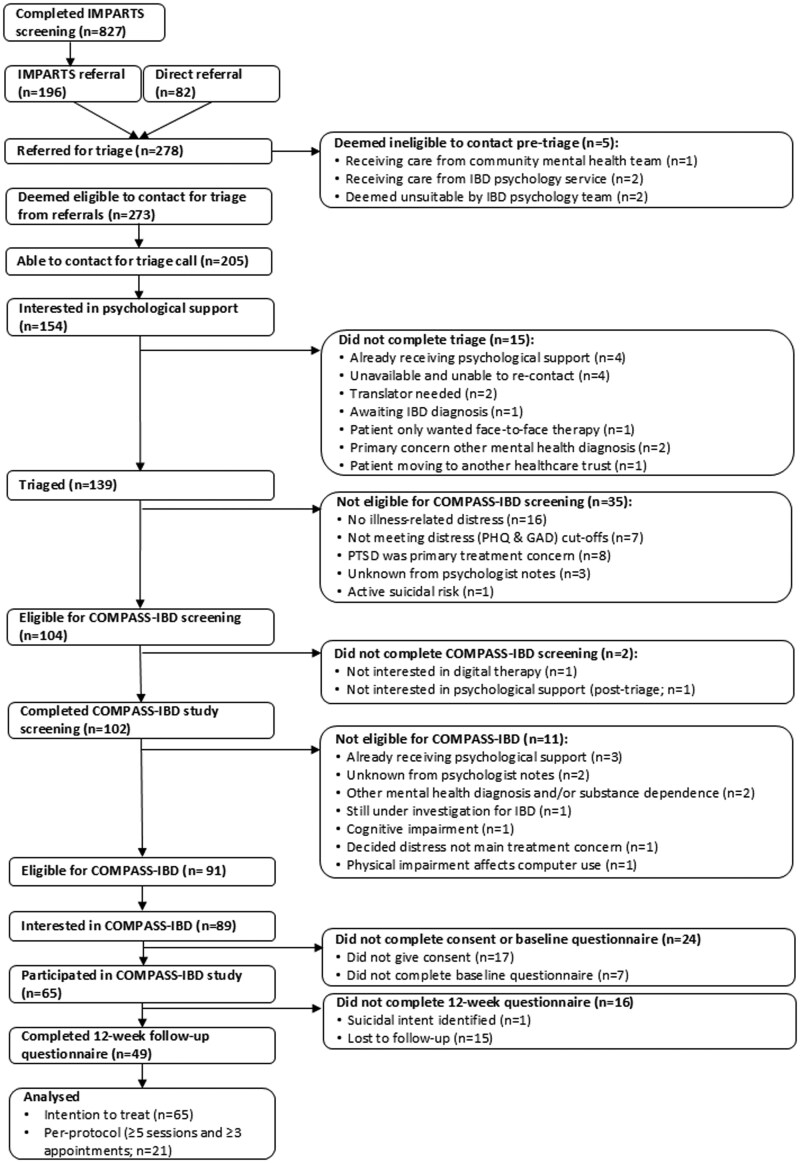
Flow of participants through the study. IBD, inflammatory bowel disease; PTSD, posttraumatic stress disorder.

Figure 1 shows the flow of participants through the implementation study and reasons for not progressing to the next stage. Table 1 summarizes the data for each of the reach objectives. The flow and sample characteristics relevant to each objective are presented in detail below.

#### Routine mental health (IMPARTS) screening

IMPARTS screening took place from November 4, 2022, to September 26, 2023. IMPARTS access was then curtailed due to a Trust-wide introduction of a new replacement electronic patient record system for managing outcome measures (Epic), which launched on October 5, 2023. Although Epic includes potential mental health screening functionality, this was not utilized by the service during study implementation; thus, screening in the current study was terminated early. Throughout the study, IMPARTS experienced multiple network failures, totaling 3 months of service (screening) disruption.

A total of 827 patients completed IMPARTS routine mental screening. The IMPARTS system itself does not provide the total number of patients contacted to complete screening. A separate system, responsible for sending the text messages with the screening link (DrDoctor; https://www.drdoctor.co.uk/), reported 4571 contacts over the study period. Given the 3-month IMPARTS disruption, we estimate that the approximate number of contacts made was 3324, meaning an estimated reach rate of 24.9% (95% CI, 23.4% to 26.4%). Near-equal numbers of men and women completed IMPARTS screening, with a mean age of 39.35 ± 14.00 years. Just over 50% of patients had missing race/ethnicity data on their electronic patient record, and 11% identified as belonging to a minority ethnic group (see Table 2 for additional demographic details).

**Table 2.** Demographic and clinical outcomes of patients completing routine mental health screening (IMPARTS) **(n = 827).**

Demographic characteristic	**n (%**)	Mean ± SD
**Age (minimum = 18 yr, maximum = 87 yr), yr**	—	39.35 ± 14.00
**Sex**		
** Female**	413 (49.9)	—
** Male**	414 (50.1)	—
**Race/ethnicity**		
** Asian or Asian British**	32 (3.9)	—
** Black or Black British**	25 (3.0)	—
**Mixed/multiple ethnic groups**	17 (2.1)	—
** Other minority ethnic groups**	16 (1.9)	—
** White**	318 (38.5)	—
** Not known**	419 (50.7)	—
**IBD diagnosis**		
** Crohn’s disease**	476 (57.6)	—
** Ulcerative colitis**	351 (42.4)	—
**Anxiety (GAD-7)**		
** Total score**	—	3.04 ± 5.10
** Below clinical cutoff**	669 (80.9)	0.79 ± 0.86
** Mild anxiety**	46 (5.6)	7.39 ± 1.22
** Moderate anxiety**	59 (7.1)	11.78 ± 1.34
** Severe anxiety**	53 (6.4)	17.98 ± 2.24
**Depression (PHQ-8)**		
** Total score**	—	3.20 ± 5.71
** Below clinical cutoff**	671 (81.8)	0.67 ± 0.87
** Mild depression**	32 (3.9)	7.59 ± 1.19
** Moderate depression**	59 (7.1)	12.27 ± 1.44
**Moderately severe depression**	38 (4.6)	16.76 ± 1.67
** Severe depression**	27 (3.3)	21.81 ± 1.66
**Distress (PHQ-ADS)**		
** Total score**	—	6.24 ± 10.07
** Below clinical cutoff**	646 (78.1)	1.68 ± 2.04
** Mild distress**	88 (10.6)	13.58 ± 2.70
** Moderate distress**	44 (5.3)	24.30 ± 3.09
** Severe distress**	49 (5.9)	37.00 ± 5.01

Abbreviations: IBD, inflammatory bowel disease; GAD, Generalized Anxiety Disorder Questionnaire; PHQ, Patient Health Questionnaire, 8-item version; PHQ-ADS, Patient Health Questionnaire Anxiety and Depression Scale.

In the sample who completed prebaseline IMPARTS screening, 18.2% (95% CI, 15.5% to 21.1%) scored over clinical cutoffs for depressive symptomology, 19.1% (95% CI, 16.5% to 22.0%) scored over clinical cutoffs for anxious symptomology, and 21.8% were over the clinical cutoff for distress on the PHQ-ADS (95% CI, 19.0% to 24.7%). In total, 26.0% (95% CI, 23.0% to 29.1%) screened positively, meeting clinical cutoffs for either depressive or anxious symptomology. Given that the outcome variables were positively skewed, binary logistic regression models were used to examine the relationships in the prebaseline IMPARTS screening sample between age, sex, race/ethnicity, and IBD diagnosis with clinical caseness for the outcomes (PHQ-ADS, GAD-7, PHQ-8) and any relevant interaction effects. Likelihood of meeting anxiety cutoffs was significantly associated with female sex (odds ratio [OR], 0.70; *P = *.05) and belonging to a minority ethnic group (OR, 2.06; *P = *.02). Odds of meeting depression cutoffs were greater for those who had a CD diagnosis compared with UC (OR, 0.64; *P = *.02). Gender interacted significantly with IBD subtype for all variables. For men, having a CD diagnosis increased the likelihood of meeting clinical cutoffs for distress (OR, 0.47; *P = *.03) and depression (OR, 0.38; *P = *.01); however, in women, there appeared to be no difference in rates between CD and UC. Women had an increased incidence of clinical anxious symptomology if they had UC, but men had an increased incidence of anxious symptomology if they had CD (OR, 0.36; *P < *.01).

#### Triage and COMPASS-IBD screening

Triage and COMPASS-IBD screening calls took place between November 2022 and October 2023. Figure 1 shows the flow of patients through triage and screening processes, including reasons for exclusion or ineligibility at each stage of the process. A greater proportion of patients met anxious symptomology cutoffs who were referred directly (94%) vs the IMPARTS sample (73%), whereas the reverse was true for depressive symptomology cutoffs (direct referral = 66%; IMPARTS referral = 75.3%). Proportions were similar for those meeting distress cutoffs between referral routes (direct referral = 86%; IMPARTS referral = 89.10%). Nine patients were referred for screening and triage more than once, with a maximum number of 3 repeat referrals (n = 1). In the 139 participants who completed triage, mean GAD-7 scores were 11.51 ± 5.56, mean PHQ-8 scores were 11.17 ± 7.45 (indicating clinical levels of depressive and anxious symptomology), and 66 of 139 (47.5%; 95% CI, 39.0% to 56.1%) patients scored above 0 on the Primary Care PTSD Screen for DSM-5 at screening, indicating some level of PTSD symptomology (mean 2.49 ± 1.67). A total of 65 of 91 (71.4%; 95% CI, 61.0% to 80.4%) eligible patients participated in the COMPASS-IBD study, meeting the green RAG rating.

#### COMPASS-IBD intervention uptake

Table 3 describes the demographic and clinical characteristics of the COMPASS-IBD sample. Most participants were female, White, employed, and with tertiary-level education. A diagnosis of CD was reported by 64% of the sample, and the average length of IBD diagnosis was 9.67 ± 9.78 years. The most common comorbidities in the sample were depression (n = 22 of 65 [33.8%]) and anxiety, respectively (n = 18 of 65 [27.7%]). There were no significant differences between the IMPARTS and COMPASS-IBD samples for age, sex, race/ethnicity, and IBD subtype.

**Table 3.** Demographic and clinical characteristics of participants in the COMPASS-IBD study (N = 65).

Demographic characteristic	n (%)	Mean ± SD
Clinical characteristic		
**Age (minimum = 18 yr, maximum = 74 yr), yr**	—	36.49 ± 13.62
**Sex**		
** Female**	38 (58.5)	—
** Male**	26 (40)	—
** Other (trans female)**	1 (1.5)	—
**Race/ethnicity**		
** Asian or Asian British**	7 (10.8)	—
** Black or Black British**	4 (6.2)	—
** Mixed/multiple ethnic groups**	3 (4.6)	—
** Other ethnic groups**	2 (3.1)	—
** White**	49 (75.4)	—
**Referral route**		
** IMPARTS screening**	45 (69.2)	—
** Direct referral**	20 (30.8)	—
**Index of Multiple Deprivation Decile (minimum = 1, maximum = 10)^a^**	—	5.13 ± 2.38
**Employment status**		
** Employed**	44 (67.6)	—
** Unemployed**	4 (6.2)	—
** Retired**	4 (6.2)	—
** Student**	4 (6.2)	—
** Long-term sick or disabled**	5 (7.7)	—
** Homemaker/carer**	1 (1.5)	—
** Not working and not on benefits**	3 (4.6)	—
**Education**		
** Secondary (GSCE, A-Level or equivalent)**	15 (23.1)	—
** Tertiary (undergraduate degree, diploma, or equivalent course)**	47 (72.3)	—
** No education**	3 (4.6)	—
**Living situation**		
** Living alone**	8 (12.3)	—
** Living with partner and/or children/family**	38 (58.5)	—
** Living with parents**	11 (16.9)	—
** Living with nonrelatives**	8 (12.3)	—
**IBD diagnosis**		
** Crohn’s disease**	42 (64.4)	—
** Ulcerative colitis**	19 (29.2)	—
** Indeterminate**	3 (4.6)	—
** Unsure**	1 (1.5)	—
**Years since IBD diagnosis (minimum = 0, maximum = 45)**	—	9.67 ± 9.78
**Number of people reporting at least 1 comorbidity**	21 (32.3)	—
**Comorbidities^b^**		
** Anxiety disorder**	18 (27.7)	—
** Asthma**	7 (10.8)	—
** Atrial fibrillation**	1 (1.5)	—
** Chronic pain**	7 (10.8)	—
** Cognitive and learning disabilities**	2 (3.1)	—
** Coronary heart disease**	1 (1.5)	—
** Depression**	22 (33.8)	—
** Diabetes**	1 (1.5)	—
** Hypertension**	3 (4.6)	—
** Liver disease**	1 (1.5)	—
** Lupus**	1 (1.5)	—
** Obesity**	2 (3.1)	—
** Osteoarthritis**	4 (6.2)	—
** Osteoporosis**	2 (3.1)	—
** Rheumatoid arthritis**	1 (1.5)	—
** Other^c^**	10 (15.4)	—
**Use of psychotropic medication**		
** Prescribed and taking**	18 (27.7)	—
** Prescribed and not taking**	6 (9.2)	—
** Not prescribed**	40 (61.5)	—
** Unsure**	1 (1.5)	—
**Anxiety (GAD-7)**		
** Total score**	—	11.60 ± 5.07
** Below clinical cutoff**	5 (7.7)	3.00 ± 1.23
** Mild anxiety**	20 (30.8)	7.60 ± 1.43
** Moderate anxiety**	24 (36.9)	12.00 ± 1.41
** Severe anxiety**	16 (24.6)	18.69 ± 1.92
**Depression (PHQ-9)**		
** Total score**	—	11.95 ± 6.23
** Below clinical cutoff**	7 (10.8)	2.57 ± 0.98
** Mild depression**	20 (30.8)	7.35 ± 1.35
** Moderate depression**	15 (23.1)	11.33 ± 1.35
** Moderately severe depression**	13 (20.0)	17.38 ± 1.61
** Severe depression**	10 (15.4)	21.60 ± 1.71
**Distress (PHQ-ADS)**		
** Total score**	—	23.55 ± 10.23
** Below clinical cutoff**	4 (6.2)	6.50 ± 0.58
** Mild distress**	21 (32.3)	15.24 ± 3.24
** Moderate distress**	22 (33.8)	23.45 ± 2.96
** Severe distress**	18 (27.7)	37.17 ± 4.60

Abbreviations: IBD, inflammatory bowel disease; GAD, Generalized Anxiety Disorder Questionnaire; PHQ-9, Patient Health Questionnaire, 9-item version; PHQ-ADS, Patient Health Questionnaire Anxiety and Depression Scale.

a n = 60, as 5 participants’ postcodes had no socioeconomic status data available.

b Each comorbidity percentages reflect frequency out of the total sample (n = 65).

c Acute back pain, cervical spondylosis, fibromyalgia, hernia, albinism, coeliac disease, sleep apnea, anemia, insomnia, heart failure, Gilbert’s syndrome, functional neurological disorder, hypothyroidism, chronic obstructive pulmonary disease, lung cancer, and myocarditis.

Baseline outcomes of the COMPASS-IBD sample are summarized in Table 3. Mean scores on the PHQ-9, GAD-7, and PHQ-ADS were above moderate clinical cutoffs. The severe cutoffs on the GAD-7 and PHQ-ADS and moderately severe-to-severe cutoffs on the PHQ-9 were met by 25%, 27.7%, and 58.5% of the sample, respectively. Nine people scored positively on PHQ-9 item 9 (n = 9 of 65 [13.8%]), indicating suicidal ideation. Baseline levels of other potential effectiveness outcomes can be seen in Table 4.

**Table 5.** Engagement and adherence variables for COMPASS-IBD.

Engagement	Data	95% CI
**COMPASS-IBD digital sessions completed (minimum = 0, maximum = 11)**	3.83 ± 3.64	2.93-4.73
**Total time spent on COMPASS-IBD, min**	59 (6-136)	
**Digital onboarding required, %**	2 (3.1)	0.4-10.7
**Therapist appointments attended (minimum = 0, maximum = 8)**	3.28 ± 2.32	2.70-3.85
**Appointment time (minimum = 0, maximum = 360), min**	142.71 ± 103.61	117.04-168.38
**Appointment format**		
** Videoconferencing, %**	67 (78.4)	72.3-83.7
** Telephone, %**	46 (21.6)	16.3-27.7
** Message, %**	0 (0.0)	0-1.7
**Adherence^a^**		
**Nonengagers (never logged on), %**	11 (16.9)	8.8-28.3
**Dropouts (disengaged with platform after initial use), %**	11 (16.9)	8.8-28.3
**Adherent to appointment component (≥3 therapist appointments), %**	43 (66.2)	53.4-77.4
**Adherent to online component (≥5 online sessions), %**	23 (35.4)	23.9-48.2
**Fully adhered (≥3 therapist appointments or equivalent and ≥5 online sessions), %**	21 (32.3)	21.2-45.1

Values are mean ± SD, median (interquartile range), or n (%).

Abbreviation: CI, confidence interval.

a Adherence categories do not sum to 100%, as fully adhered participants are counted in proportions for the separate adherence components.

**Table 4.** Treatment effect estimates and standardized mean difference for effectiveness adjusted for age, sex, race/ethnicity, and common mental disorder diagnosis at baseline (N = 65).

	Baseline	12 wk^a^	B	SE	95% CI	*P*	Cohen’s *d*	SRM
**Distress (PHQ-ADS)**	23.55 ± 10.23	16.19 ± 11.26	−6.448	1.30	−8.98 to −3.91	<.001^b^	−0.573	−0.677
**Anxiety (GAD-7)**	11.60 ± 5.07	7.82 ± 5.67	−3.533	0.69	−4.89 to −2.18	<.001^b^	−0.627	−0.690
**Depression (PHQ-9)**	11.95 ± 6.23	8.37 ± 6.08	−2.913	0.73	-4.34 to −1.49	<.001^b^	−0.456	−0.552
**PGIS**	2.25 ± 0.97	2.10 ± 1.02	−0.122	0.15	−0.42 to 0.17	.417	−0.124	−0.121
**IBD-related Quality of life (IBDQ)**	80.25 ± 15.93	87.34 ± 17.89	7.078	1.89	3.37 to 10.79	<.001^b^	−0.400	−0.565

Values are mean ± SD, unless otherwise indicated. The Patient Global Impression of Improvement Scale (PGII) was listed as an effectiveness outcome; however, due to technical issues with the survey software, it was not collected.

Abbreviations: CI, confidence interval; GAD, Generalized Anxiety Disorder Questionnaire; IBD, inflammatory bowel disease; IBDQ, Inflammatory Bowel Disease Questionnaire; PGIS, Patient Global Impression of Severity Scale; PHQ-9, Patient Health Questionnaire, 9-item version; PHQ-ADS, Patient Health Questionnaire Anxiety and Depression Scale; SRM, standardized response mean.

a 12-week data presented only for those who responded (Missing Completely At Random [MCAR] assumption), even though the mixed model is based on a Missing At Random (MAR) assumption.

b *P *≤ .001.

c *P *≤ .01.

d *P *≤ .05.

### Implementation

#### COMPASS-IBD adherence and engagement

Engagement and adherence data for the digital and therapist components for COMPASS-IBD are presented in Table 5.

Two COMPASS-IBD patients requested digital onboarding support, which took on average 20 ± 14.14 minutes of therapist time. Patients spent a median of 59 minutes (interquartile range 6-136 minutes) on the COMPASS-IBD online platform and completed a mean of 3.83 ± 3.64 sessions. Participants attended a mean of 3.28 ± 2.32 therapist appointments, averaging 142.71 ± 103.61 minutes spent with COMPASS therapists. Most sessions were conducted via video call.

Of the 65 participants, 43 (66.2%; 95% CI, 53.4% to 77.4%) adhered to the appointment component (≥3 therapist appointments) and 23 (35.4%; 95% CI, 23.9% to 48.2%) adhered to the online component (≥5 online sessions). Eleven (16.9%; 95% CI, 8.8% to 28.3%) did not engage in the COMPASS-IBD treatment at all, and 11 (16.9%; 95% CI, 8.8% to 28.3%) dropped out after initial use. The composite measure of adherence (≥3 therapist appointments and ≥5 online sessions) was met by 21 patients (32.3%; 95% CI, 21.2% to 45.1%), signifying a red RAG rating.

### Acceptability

#### Patient intervention acceptability

Based on the TFA questionnaire,^25^ mean acceptability of the intervention was 23.15 ± 4.74 of 30. Between 55% and 75% of participants rated “agree” or “strongly agree” on the 6 constructs (affective attitude: 67.5% burden: 60.0%; perceived effectiveness: 55.0%; ethicality: 72.5%; intervention coherence: 75.0%; self-efficacy: 65.0%) (Appendix 4 shows graphs representing all ratings).

### Potential effectiveness outcomes

#### Patient level

##### Primary and secondary effectiveness outcomes

**Figure 2.**
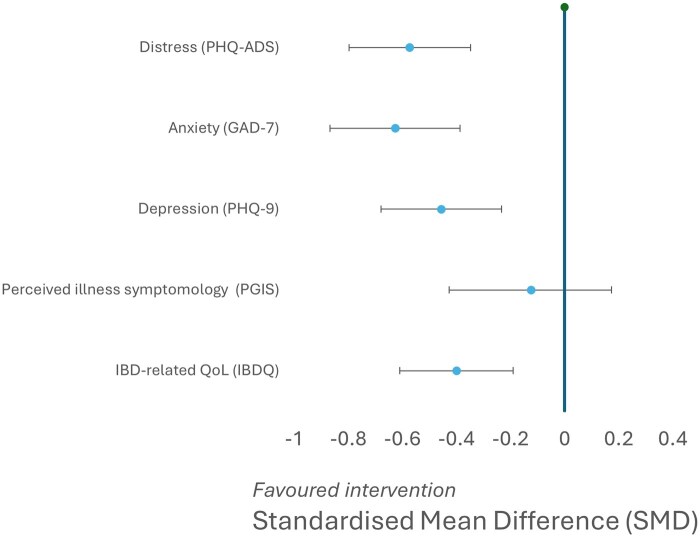
Forest plot of adjusted treatment effects for primary and secondary effectiveness variables. The PGI-I was listed as an effectiveness outcome; however, due to technical issues with the survey software, it was not collected. Error bars represent 95% confidence intervals. GAD, Generalized Anxiety Disorder Questionnaire; IBD, inflammatory bowel disease; IBDQ, Inflammatory Bowel Disease Questionnaire; PGI-I, Patient Global Impression of Improvement Scale; PGIS, Patient Global Impression of Severity Scale; PHQ-9, Patient Health Questionnaire, 9-item version; PHQ-ADS, Patient Health Questionnaire Anxiety and Depression Scale; QoL, quality of life.

The adjusted treatment effects of COMPASS-IBD for the primary and secondary effectiveness outcomes are presented in Table 4 (see Figure 2 for a forest plot of treatment effects).

There was a significant reduction in the primary outcome distress of −6.448 (95% CI, −8.98 to −3.91; *P < *.001) on the PHQ-ADS, with a medium Cohen’s *d* effect size of −0.573 from pre- to post-intervention. This reduction is 61% larger than the minimum clinically important difference for the PHQ-ADS (≥4), thus signifying a green RAG on the potential effectiveness progression criterion.

Regarding secondary outcomes there were significant moderate effects for anxious symptomology (GAD-7: −3.533; 95% CI, −4.89 to −2.18, *P < *.001; Cohen’s *d = −*0.627), and significant small-moderate effects for depressive symptomology (PHQ-9: −2.913; 95% CI, −4.34 to −1.49; *P < *.001; Cohen’s *d = −*0.456) and IBD-related quality of life (IBDQ: 7.078; 95% CI, 3.37 to 10.79; *P < *.001; Cohen’s *d *= 0.400). There was no significant effect for illness symptomology (PGIS: −0.122; 95% CI, −0.42 to 0.17; *P = *.417; Cohen’s *d = −*0.124). Due to a technical error, the Patient Global Impression of Improvement (PGI-I) scale (meant to be measured post-intervention only) was not collected. A per-protocol sensitivity analysis was conducted in fully adherent patients. The effects were generally consistent with the intention-to-treat analysis (see Appendix 5). There were no significant modifiers of treatment effect, although a trend was observed for race/ethnicity, whereby ethnic minority groups had smaller reductions in distress (B = 5.06; *P = *.08) (see Appendix 6).

##### Putative mediators

Appendix 7 shows adjusted treatment effects for the proposed treatment effect mechanisms (see Appendix 8 for a forest plot). Participants demonstrated significantly improved cognitive and behavioral responses to symptoms (eg, reduced embarrassment avoidance and all-or-nothing behavior subscale scores), demonstrated increased acceptance of chronic disease, and had less negative illness perceptions (using a sum score with the Brief Illness Perception Questionnaire as per previous research). Some self-efficacy subscales also significantly improved (general self-efficacy, depression management), but others showed no change (exercise, obtaining help, social, managing symptoms, obtaining disease information).

#### Service level

##### COMPASS-IBD waitlist change

Before the start of the study, at an equivalent time period (November 2021 to November 2022), the waitlist for IBD psychology was 11.95 months (95% CI, 8.38 to 15.52 months) long, and the number of patients on the waitlist was 120. After study inception, the waitlist initially increased to a maximum of 216 patients (waitlist time increase at this stage unknown); however, by the end of the study, the waitlist number decreased to 79 patients, and the waitlist time decreased to 8.27 months (95% CI, 6.94 to 9.60 months) long. During the study, to increase support provision, 2 additional external HCPs (1 health psychologist [paid by the research project for their time], 1 CBT-trained honorary assistant psychologist [a volunteer position supporting a registered psychologist with clinical tasks and research]) were trained to deliver COMPASS-IBD. These two additional HCPs supported 14 patients through the COMPASS-IBD program.

##### COMPASS-IBD discharge outcome

Twenty-eight patients who were on the IBD psychology ­waitlist while completing COMPASS-IBD were able to be discharged from the IBD psychology service postcompletion. Sixteen patients were “stepped-up” and added to the waitlist for further support postcompletion. The remaining 21 patients had been on the waitlist prior to COMPASS-IBD and wished to remain on the waitlist to receive additional treatment after completing COMPASS-IBD. Generally, reasons for being added to or remaining on the waitlist after COMPASS-IBD were continued high levels of distress, clinical complexity (eg, experiencing PTSD symptoms), and desiring further support or a different treatment modality (eg, one-on-one or group therapy).

## Discussion

The COMPASS-IBD study aimed to evaluate the real-world implementation of a novel digital pathway to identify and treat illness-related distress in a large gastroenterology service, with a view to determining progression to a larger-scale hybrid implementation study across multiple services. This article focused on understanding the reach, implementation, acceptability and effectiveness of the new integrated pathway (utilizing digital mental health screening and the digital CBT program COMPASS-IBD) in reducing psychological distress for people living with IBD. This includes assessing patient outcomes in regards to effectiveness and acceptability, and more broadly investigating the feasibility of delivering the new treatment pathway via reach and implementation outcomes. Using our predefined RAG criteria, the study was deemed eligible to proceed to a large implementation study on both (1) reach (participation rate), with 71% of eligible participants consenting to take part in the COMPASS-IBD intervention; and (2) effectiveness, with a mean change on the primary outcome (PHQ-ADS) of ≥4 observed. However, implementation of predefined RAG criteria was not met, as only one-third of participants met our composite adherence measure to both online and therapist appointments. However, it is worth noting that the acceptability ratings of the intervention were good. This, alongside the intention-to-treat effectiveness data, suggests the adherence cutoff may have been set high, although it still needs improving. The possible reasons for the low level of adherence and strategies to address it prior to launching a full-scale hybrid type II trial are discussed subsequently, as are the detailed data relating to the other broader objectives.

In regards to our Reach objective, IMPARTS mental health screening was completed by 827 patients (an estimated 24.9% of patients presenting to the clinic). Of the patients screened, 19.1%, 18.9% and 21.8% demonstrated at least mild symptomology in relation to anxiety, depression, and distress, respectively. This is significant given that this distress would not have been highlighted without our new integrated pathway. These rates of distress are similar to those previously observed in other IBD populations, including a recent meta-analysis (25.3% for depression and 32.1% for anxiety).^3^ The slightly lower rates in the current study may be due to differences in measurement, as participants only proceeded to complete the full PHQ and GAD measures if they met initial score criteria on the PHQ-2 and GAD-2 initial items. Although this is intended to reduce participant burden, this method has decreased sensitivity for capturing distress in IBD ­populations,^33^ as those who may exhibit distress in the full measure are not captured.

There were several implementation challenges with the IMPARTS software used for screening, including a 3-month service failure period. Race/ethnicity data were not entered for 51% of patients due to missing data on patient records, limiting our understanding of ethnic-level differences in screening completion. However, the ethnic breakdown observed in our study generally aligns with the service’s catchment area (Lambeth, Southwark, and Kent; see Appendix 9), suggesting that individuals in the screened sample were representative of the target population.

Despite the digital challenges, the inclusion of standard digital screening did improve the recognition of psychological distress within the service. During the study, 196 patients met criteria for triage and were referred to the psychology team through the new digital screening. This suggests that the pathway better captured patient need for psychological support in the IBD service, which led to an initial increase in the number of patients on the IBD psychology waitlist. Importantly, by the end of the study, the waitlist number and time reduced (by 63.4% and 30.8%, respectively) as compared with preimplementation, suggesting that over time the pathway was efficiently integrated into the IBD psychology service. However, it is important to note that the research team provided admin and screening support to facilitate the new integrated pathway. Future implementation in routine care would therefore require consideration of how this administrative support could be facilitated within the IBD service.

Of those patients identified through IMPARTS screening or direct referral, 154 were interested in receiving psychological support, with 71.4% of eligible participants progressing through to the COMPASS-IBD program. This compares favorably to similar studies implementing psychological screening and treatment into IBD secondary care services, observing a 51% treatment uptake rate.^8^ Importantly, 47.5% of those ­triaged exhibited PTSD symptoms related to their IBD. It is important that future work considers possible scalable interventions for supporting PTSD symptoms in IBD as well. Moderation analyses revealed some differences in distress between disease type, sex, and race/ethnicity. With regard to sex, these differences were novel, potentially reflecting sex-based differences in IBD pathogenesis and presentation.^34^^,^^35^ The incidence of particular comorbidities and extraintestinal manifestations varied significantly between men and women.^35^ Men with CD have an increased likelihood of more extensive and complicated disease, as well as colorectal cancer. Women with IBD report higher pain and fatigue, potentially due to female sex hormones influencing immune function and symptom presentation.^34^ Women additionally report more sexual dysfunction than men, with particularly high prevalence in women with UC.^36^ Coping strategies can also differ between men and women,^37^ with women demonstrating increased problem- and emotion-focused coping. However, these findings warrant further exploration in larger, more representative IBD samples.

Regarding our implementation objective, as noted previously, the RAG criterion for adherence was not met. Our composite measure set a minimum of 5 digital sessions; however, patients were not advised on a set number of sessions to complete, and could choose which content to engage with however they liked (eg, they may have spent more time on certain sessions while choosing not to complete others). On average, patients completed 4 sessions, which may have been sufficient for their needs. More work is needed to define an optimal number of sessions in relation to outcome for COMPASS, to provide patients with clearer targets.

Additionally, around one-third of patients did not engage at all with COMPASS-IBD postenrollment or dropped out. While reasons for dropout were not formally captured, some participants disclosed that illness flares and symptom burden affected their ability to engage. Attrition has been found to vary substantially across online therapy interventions in IBD, with similar interventions reporting 26% to 91% adherence.^38^^,^^39^ Future work should focus on understanding reasons for disengagement. Modifications to enhance engagement might include making greater use of multimedia for content or incorporating additional touch-point phone calls. Patient expectations are also related to treatment initiation and attendance^40^; therefore, providing more information on how and why COMPASS-IBD works, or targeting patients interested in digital therapy, may also improve future engagement. It is also worth noting that the average number of sessions completed and session length were similar between the online content and therapist support sessions, suggesting that the “guided” aspect of COMPASS may be particularly important for patient engagement. Future research could explore this further and evaluate the effectiveness of different COMPASS-IBD delivery methods, such as self-led vs guided support.

Over two-thirds of our sample were adherent to the therapist component of COMPASS-IBD, similar to rates of face-to-face therapy reported in previous integrated care pathways (67%).^8^ This rate is very acceptable considering that COMPASS-IBD therapy was delivered by trainee therapists, who were new to working in gastroenterology and were only there on a 6-month placement. In terms of the acceptability rating on the TFA, most patients found COMPASS-IBD therapy as a whole (digital plus therapist) to be an acceptable treatment. However, certain aspects of acceptability, such as the intervention being an acceptable burden, were rated lower than others, suggesting that products that are less time-intensive or have less content may be preferred by some patients. In terms of the effectiveness objective, COMPASS-IBD showed moderate effect sizes for reductions in psychological distress in the intention-to-treat analysis. This corresponded to a 6-point reduction, which exceeds the minimal clinically important difference on the PHQ-ADS of 4.^23^ Significant effects were also observed for improvements in anxious and depressive symptomology, and IBD-related quality of life. These improvements are consistent with the original COMPASS RCT in patients with 4 different LTCs, including IBD.^13^ The effect size improvements in the current study are better than those observed in a real-world study of COMPASS implemented in NHS mental health Talking Therapies services (formerly known as Improving Access to Psychological Therapies).^11^ The larger effects seen from COMPASS-IBD may be due to the tailoring of content specifically to the concerns of IBD, thus increasing relevance and acceptability of the intervention.^22^^,^^32^ It may also be to do with the current study setting. Talking Therapies is a generic mental health service. Therapists in these settings primarily diagnose and treat primary mental health concerns, as opposed to distress related to living with an LTC. Psychological therapy integrated into secondary care services may enhance the treatment coherence (reflected in the current study by high patient ratings on the coherence component of the TFA).

In terms of service-level effectiveness, 28 patients were able to be discharged from the IBD psychology waitlist following completion of COMPASS-IBD. This suggests that COMPASS-IBD alone may be a sufficient intervention for patients referred for support who are experiencing milder levels of distress.

This was the first study to date to explore the effects on the hypothesized treatment mechanisms on which COMPASS-IBD therapy was designed. There were small effects on most of the variables hypothesized to be key mechanisms of change in COMPASS-IBD, including reduced embarrassment avoidance and all-or-nothing behavior, improved illness perceptions, increased acceptance of chronic disease, and improved self-efficacy for disease management. A future randomized controlled trial is needed to assess if these are indeed mediators of the treatment effect.

### Strengths and limitations

This study was embedded within an NHS gastroenterology clinic and consequently provides insight into the feasibility, acceptability, and effectiveness of an integrated care pathway in a real-world setting. Although the sample was ethnically diverse and scored close to the national median social deprivation index, people with higher education levels were overrepresented, which may limit the generalizability of the findings. Moreover, due to the digital nature of the IMPARTS and COMPASS-IBD programs, elderly or socioeconomically disadvantaged groups (such as those with lower education or ethnic minorities) may have been less likely to access and benefit from the new integrated pathway. This may have been further exacerbated by the technological issues, which interrupted access to IMPARTS and potentially patient engagement in future screening. The absence of a control group within the study limits the extent to which psychological improvements can be attributed to COMPASS-IBD. Additionally, the research team supported implementation of the new integrated pathway throughout this study, including 2 additional therapists utilized to increase intervention provision. While this was necessary to conduct the study, these resources were provided by the current research project and funding (eg, research team full time equivalent (FTE)). Future implementation should assess how the pathway could function independently within services to inform an economic evaluation of the cost-effectiveness of the pathway. Trainee therapists delivered the intervention, and these may not be available to all gastroenterology services. Additionally, while all therapists received standard supervision as per-service protocols, therapist adherence to the protocol or therapist effects were not measured in the current study given we were focused on outcomes related to implementation in routine care. Finally, the use of real-world data introduced challenges with data collection for certain outcomes, due to restrictions in data capture and reporting at a service level (eg, data on number of patients sent IMPARTS screening).

## Conclusions and Future Directions

The current findings suggest that with some minimal extra resource (eg, trainee psychologists, admin support), digital screening and treatment for IBD-related psychological distress can be incorporated as part of routine secondary IBD care, to effectively identify and treat psychological distress. The pathway was feasible, effective, and largely acceptable to patients (HCP acceptability will be reported separately).^32^ Given the need for scalable integrated psychological care in IBD, this new pathway may help to improve mental health provision at low cost to services. Requirements for adherence to the digital component of COMPASS-IBD should be addressed before progressing to a larger multicenter implementation study. A variety of geographic locations should also be included to assess widespread implementation and generalizability of findings. ­Collection of longer-term outcomes could also assess the sustainability of pathways, intervention effects, and whether this integrated care pathway can also improve physical health and reduce healthcare utilization in IBD.

## Supplementary Material

izaf259_Supplementary_Data

## Data Availability

The data underlying this article will be shared on reasonable request to the corresponding author.
