# Supplementary material for: A Real-World Longitudinal Case-Study Implementing Digital Screening and Treatment for Distress in Inflammatory Bowel Disease: The COMPASS-IBD Patient Journey
Source: Inflamm Bowel Dis. 2026 Jan 13;32(4):620–33. doi: 10.1093/ibd/izaf259 (PMC13046055; doi:10.1093/ibd/izaf259)
Supplement: izaf259_Supplementary_Data [file izaf259_supplementary_data.zip › COMPASS-IBD_supplementarymaterial_R&R#2_290925_CLEAN.docx]

# Supplementary material

### Appendix 1. Outline of standard care pathway and the new integrated treatment pathway.

***
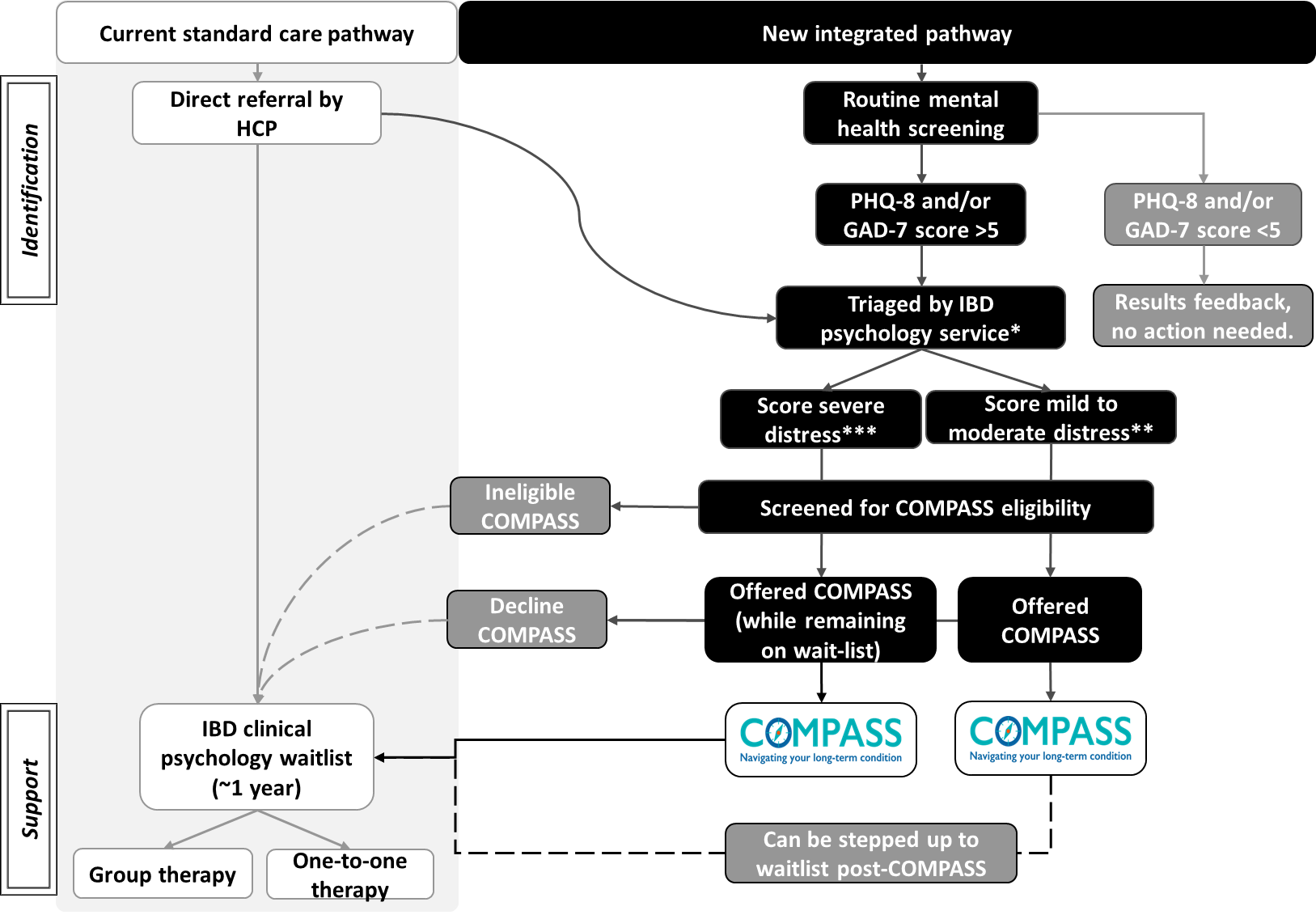
***

*NOTE.* ^*^Full triage process outlined in Appendix 2. Those directly referred who score < 5 on either the PHQ-8 or GAD-7 during triage will be offered to be referred to the IBD psychology waitlist; **mild to moderate distress is scores between 6 and 14 on the GAD-7, or between 6 to 19 on the PHQ-8; ***severe distress are scores ≥ 15 on the GAD-7, and scores ≥ 20 on the PHQ-8; PHQ= Patient Health Questionnaire, GAD= Generalised Anxiety Disorder scale.

### Appendix 2. Triage and screening full in new integrated pathway.

***
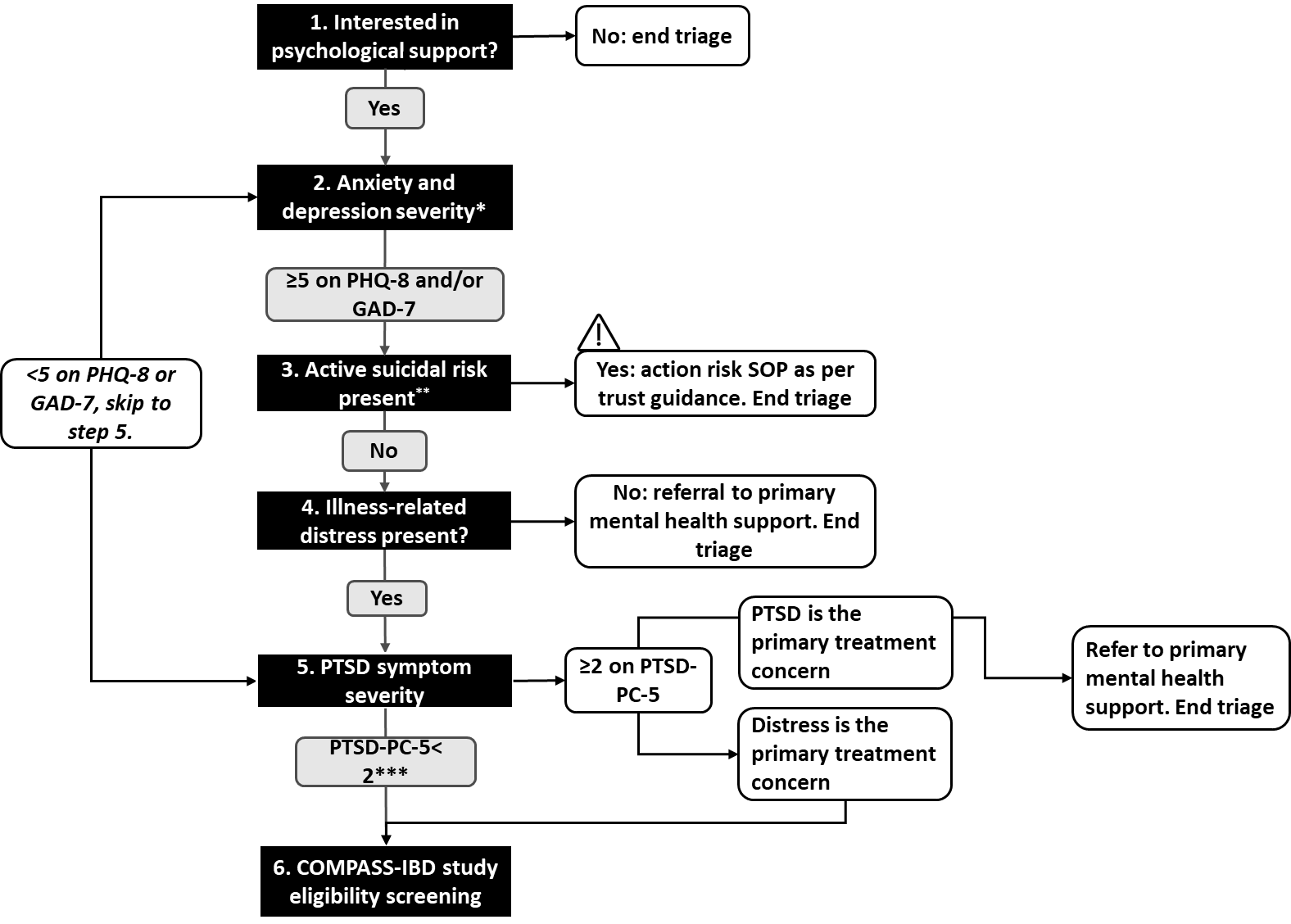
***

*NOTE****.*** *only if IMPARTS has not been completed; ^**^active risk screening completed if PHQ-8 ≥10; ***if score <2 on PTSD-SD-5 and <5 on PHQ-8 and/or GAD-7, refer to charity support; GAD= Generalised Anxiety Disorder scale; PHQ= Patient Health Questionnaire, PTSD-PC-5= Primary Care PTSD Screen, SOP= Standard Operating Procedure.

### Appendix 3. COMPASS-IBD measures mapped to study objectives.

| **Related study objective** | **Outcome** | **Operationalisation** | **Measurement** | **Data collection point** | | | **RAG criteria** |
| --- | --- | --- | --- | --- | --- | --- | --- |
|  |  |  |  | **Screening and triage** | **Pre-intervention** | **12-week (post-intervention)** |  |
| **Reach** | Sociodemographic characteristics | Patient self-report | - Patient questionnaire - Mental health screening data | X | X |  |  |
|  | Clinical characteristics | Patient self-report | Patient questionnaire |  | X | X |  |
|  | Mental health screening outcomes | - PHQ-8 (1) - GAD-7 (2) | Mental health screening data | X |  |  |  |
|  | Triage outcomes | - PHQ-8 (1) - GAD-7 (2) - Active suicidal risk - PTSD-PC-5 (3) - Illness-related distress - Interest, eligibility and participation rate^$^ for COMPASS-IBD | Triage and study screening data | X |  |  | Participation rate  R: <60%  A: <70% - ≥60%  G: ≥70% |
| **Adoption** | New treatment pathway adoption in IBD service | - Change in IBD psychology wait-list duration and length* | IBD psychology service data |  | X | X |  |
| **Implementation** | COMPASS-IBD adherence | - Number and length of digital and therapist sessions completed - Mode of session delivery - Rate of non-engagers, dropouts, users adherent to online component, users adherent to therapist component, and fully adherent users^$^ - Number of patients requiring digital onboarding | COMPASS-IBD software data and reported by therapists |  |  | X | Adherence  R: <50%  A: <60% - ≥50%  G: ≥60% |
| **Acceptability** | Patient acceptability of new treatment pathways | TFA (4) | Patient questionnaire  Cronbach α=0.94 |  |  | X |  |
|  |  | Qualitative Interviews^+^ | Semi-structured interview |  |  | X |  |
|  | HCP acceptability of new treatment pathways | Qualitative interviews^+^ | Semi-structured interview |  |  | X |  |
|  |  | NoMAD and NASS-CAT adapted questionnaire (5, 6) ^+^ | HCP implementation questionnaire |  | X | X |  |
|  | Barriers and facilitators of implementation (from research team perceptive) | Mapped to NPT and ERIC (7, 8)^+^ | Implementation plan | X | X | X |  |
| **Potential effectiveness** | Psychological distress | PHQ-ADS (9) ^$,^i28 item, composite of PHQ-9 and GAD-7, with higher scores indicating greater symptoms of distress | Patient questionnaire  Cronbach α=0.94-0.90 |  | X | X | Mean change on PHQ-ADS  R: <2  A: <4 to ≥2  G: ≥4 |
|  | Depression | PHQ-9 (10), 9 item, with higher scores indicating greater symptoms of depression | Patient questionnaire  Cronbach α=0.86-0.88 |  | X | X |  |
|  | Anxiety | GAD-7 (2), 7 item, with higher scores indicating greater anxiety symptoms | Patient questionnaire  Cronbach α=0.85-0.91 |  | X | X |  |
|  | Perceived illness symptomology | PGI-S* and PGI-I^Ⴈ^ (11), single item PRO of illness severity and improvement | Patient questionnaire |  | X | X |  |
|  | IBD-related quality of life | UK-IBDQ (12); 30 items, with higher scores indicating better quality of life | Patient questionnaire  Cronbach α=0.93-0.95 |  | X | X |  |
|  | Illness perceptions | BIPQ (13) 8 items, with higher scores indicating a more threatening perception of illness | Patient questionnaire  Cronbach α=0.80 |  | X | X |  |
|  | Cognitive and behavioural responses to illness | CBRQ-short form (14); 18 items, with higher scores indicating more maladaptive responses to illness | Patient questionnaire  Cronbach α=0.82-0.88 |  | X | X |  |
|  | Self-efficacy of illness self-management | SEMCD (15) 6 items, with higher scores indicating greater self-efficacy in managing illness | Patient questionnaire  Cronbach α=0.65-0.96 |  | X | X |  |
|  | Acceptance of illness | CHCS (16); 10 items, higher totals indicate greater acceptance | Patient questionnaire  Cronbach α=0.79-0.85 |  | X | X |  |
|  | COMPASS-IBD treatment outcome | Patient stepped-up or down post-treatment | Logged by COMPASS-IBD therapist |  |  | X |  |

Note. *Only PGI-S completed at baseline, ^$^Part of composite progression criteria with Red, amber, green (RAG) ratings provided in RAG column; ^Ⴈ^Due to a technical error the PGI-I was not collected post intervention; ^+^ indicates outcome that will be reported in a separate paper. IPQ= Brief Illness Perception Questionnaire, CBRQ= Cognitive and Behavioural Responses Questionnaire, CHCS= Acceptance of Chronic Health Conditions Scale, GAD-7= Generalised Anxiety Disorder questionnaire, HCP= healthcare professional, IBD= inflammatory bowel disease, PGI-S= Patient Global Impression Scales of Severity, PGI-I= Patient Global Impression Scales of Improvement, PHQ-8= Patient Health Questionnaire 8-item version, PHQ-ADS= PHQ Anxiety and Depression Scale, PTSD-PC-5= Primary care PTSD screen, RAG= Red, Amber, Green ratings used for composite progression criteria, SEMCD= Self-Efficacy of Managing Chronic Diseases subscales, TFA= Theoretical Framework of Acceptability questionnaire, UK-IBDQ= UK IBD Quality of Life Questionnaire.

### Appendix 4. Acceptability of intervention amongst patients based on the Theoretical Framework of Acceptability

| 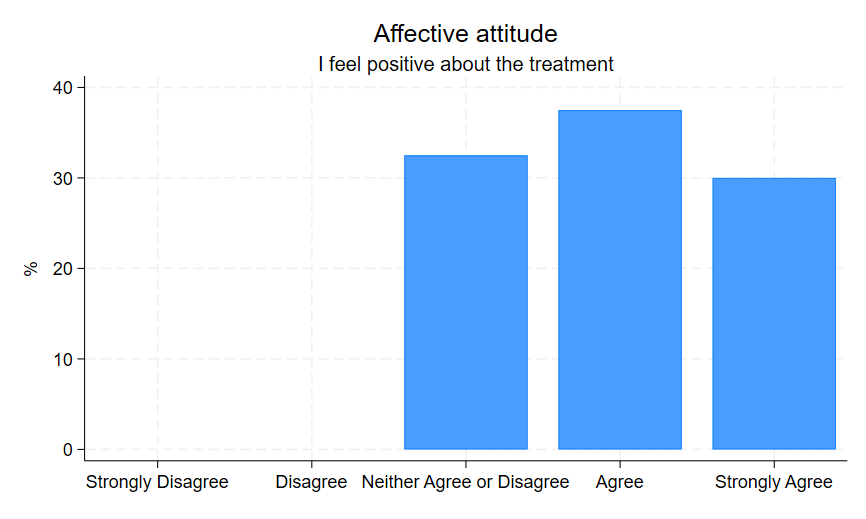 | 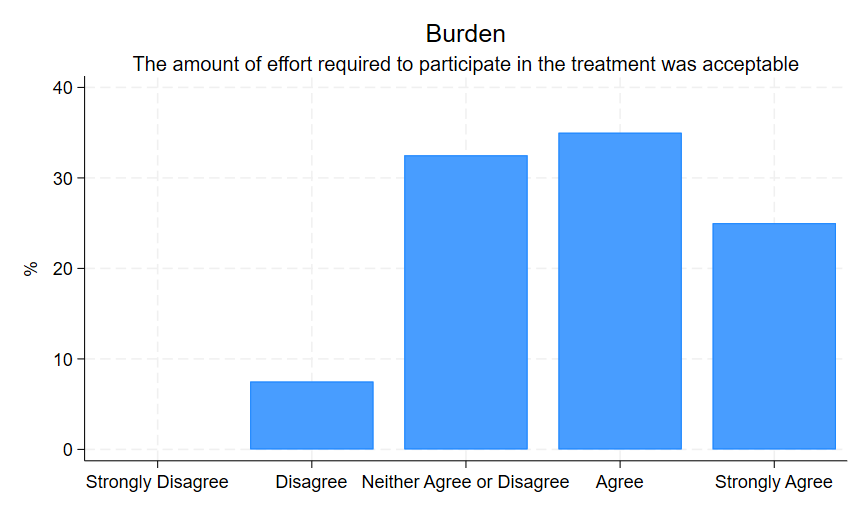 |
| --- | --- |
| 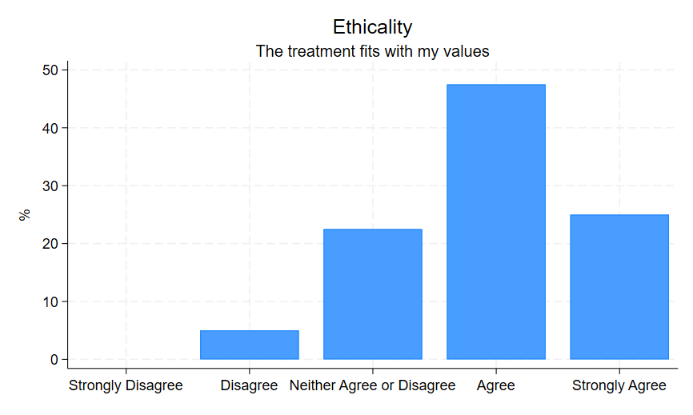 | 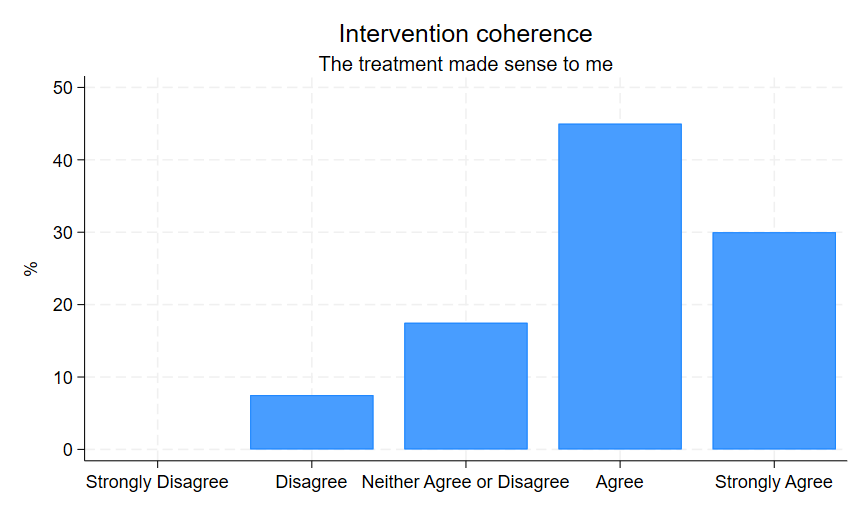 |
| 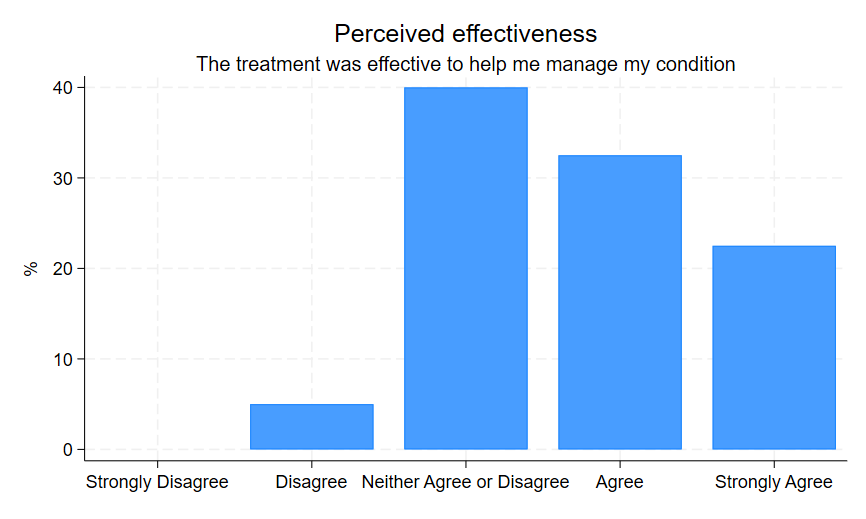 | 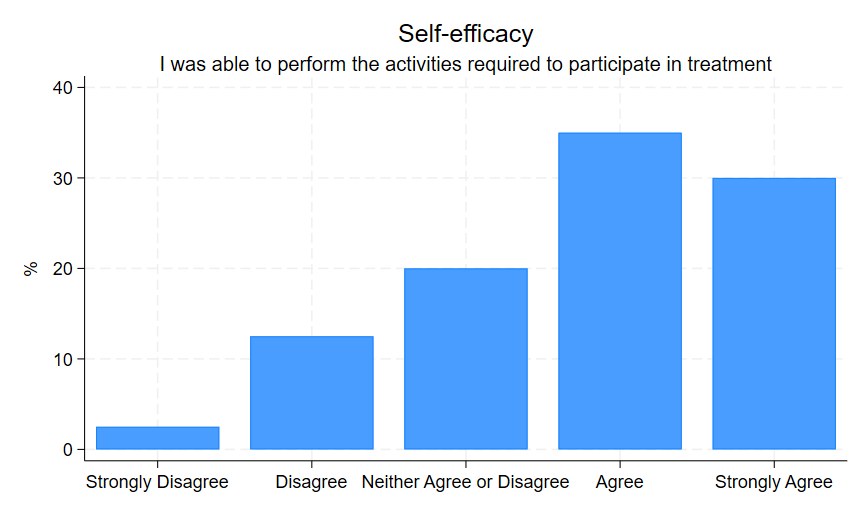 |

### Appendix 5. Sensitivity analysis for participants who fully adhered to COMPASS-IBD: treatment effect estimates and standardised mean difference for effectiveness variables

|  | n | Baseline M(SD) | 12-weeks M(SD) | B | SE | 95% CI | *p* | Cohen’s *d* | SRM |
| --- | --- | --- | --- | --- | --- | --- | --- | --- | --- |
| Distress (PHQ-ADS) | 21 | 23.38 (9.86) | 15.00 (11.23) | -8.593 | 2.33 | -13.16, -4.02 | <0.001*** | -0.764 | -0.792 |
| Anxiety (GAD-7) | 21 | 12.14 (4.98) | 7.25 (5.63) | -5.000 | 1.28 | -7.51, -2.48 | <0.001*** | -0.862 | -0.836 |
| Depression (PHQ-9) | 21 | 11.24 (5.89) | 7.25 (6.03) | -3.576 | 1.24 | -6.01, -1.14 | 0.004*** | -0.582 | -0.621 |
| PGIS | 21 | 2.10 (0.89) | 1.89 (0.88) | -0.187 | 0.17 | -0.52, 0.15 | 0.273 | -0.213 | -0.245 |
| UK IBDQ summary score | 21 | 84.63 (12.54) | 90.84 (16.54) | 6.428 | 2.14 | 2.24, 10.61 | 0.003*** | 0.435 | 0.668 |

*Note*. The PGII was listed as an effectiveness outcome, however due to technical issues with the survey software, it was not collected. GAD= Generalised Anxiety Disorder questionnaire; PGI-S= Patient Global Impression of Severity scale; PHQ= Patient Health Questionnaire; PHQ-ADS= Patient Health Questionnaire Anxiety and Depression Scale, SRM=standardised response mean, UK IBDQ= UK IBD Quality of Life Questionnaire

*** Denotes p≤0.001, ** denotes p≤0.01, * denotes p≤0.05.

### Appendix 6. Graphical representation of moderator analysis on treatment effect

|  |  |
| --- | --- |
|  |  |
|  |  |
|  |  |

Note. IMD=index of multiple deprivation, CMD=common mental disorder

### Appendix 7. Treatment effect estimates and standardised mean difference for process variables adjusted for age, gender, ethnicity and common mental disorder diagnosis at baseline

|  | n | Baseline M(SD) | 12-weeks M(SD)^$^ | B | SE | 95% CI | p | Cohen’s d | SRM |
| --- | --- | --- | --- | --- | --- | --- | --- | --- | --- |
| Embarrassment Avoidance (CBRQ) | 64 | 7.30 (3.23) | 6.24 (3.35) | -1.013 | 0.424 | -1.84, -.18 | 0.017* | -0.307 | -0.361 |
| Symptom Focusing (CBRQ) | 64 | 8.72 (2.47) | 8.61 (2.48) | -0.326 | 0.361 | -1.03, .38 | 0.365 | -0.132 | -0.137 |
| All-or-nothing behaviour (CBRQ) | 64 | 6.91 (3.29) | 5.76 (3.53) | -0.808 | 0.397 | -1.59, -.031 | 0.042* | -0.236 | -0.303 |
| Exercise Regularly (SEMCD) | 64 | 5.85 (2.75) | 6.14 (2.82) | 0.101 | 0.347 | -.58, .78 | 0.770 | 0.037 | 0.045 |
| Obtain Help From Community, Family, Friends (SEMCD) | 64 | 5.66 (1.95) | 5.96 (2.00) | 0.193 | 0.331 | -.456, .843 | 0.559 | 0.099 | 0.086 |
| Manage Disease in General (SEMCD) | 63 | 5.56 (1.9) | 5.92 (1.88) | 0.473 | 0.226 | .03, .92 | 0.037* | 0.272 | 0.319 |
| Social/Recreational Activities (SEMCD) | 63 | 5.37 (2.19) | 5.95 (2.56) | 0.516 | 0.298 | -.07, 1.10 | 0.084 | 0.221 | 0.267 |
| Manage Symptoms (SEMCD) | 63 | 4.05 (1.81) | 4.54 (2.10) | 0.368 | 0.268 | -.16, .89 | 0.169 | 0.190 | 0.205 |
| Control/Manage Depression (SEMCD) | 63 | 4.53 (1.93) | 5.38 (2.36) | 0.700 | 0.321 | .069, 1.33 | 0.030* | 0.330 | 0.319 |
| Get Information About Disease (SEMCD) | 63 | 5.69 (2.82) | 5.98 (2.45) | 0.243 | 0.340 | -.42, .91 | 0.475 | 0.091 | 0.107 |
| Illness perceptions (BIPQ total) | 64 | 6.04 (1.49) | 5.38 (1.57) | -0.603 | 0.174 | -94, -.26 | 0.001*** | -0.391 | -0.531 |
| Consequences (BIPQ) | 63 | 7.16 (2.09) | 6.35 (2.08) | -0.752 | 0.264 | -1.27, -.24 | 0.004*** | -0.356 | -0.437 |
| Personal control (BIPQ) | 63 | 3.79 (2.21) | 4.43 (2.37) | 0.697 | 0.327 | .06, 1.34 | 0.033* | 0.302 | 0.323 |
| Treatment control (BIPQ) | 63 | 6.13 (2.49) | 6.78 (2.25) | 0.366 | 0.342 | -.30, 1.04 | 0.285 | 0.149 | 0.164 |
| Identity (BIPQ) | 63 | 6.44 (2.05) | 5.58 (2.05) | -0.838 | 0.296 | -1.42, -.26 | 0.005*** | -0.403 | -0.424 |
| Concern (BIPQ) | 63 | 7.03 (2.09) | 6.70 (2.45) | -0.236 | 0.323 | -.87, .40 | 0.466 | -0.106 | -0.106 |
| Coherence (BIPQ) | 63 | 6.19 (2.46) | 6.65 (2.59) | 0.507 | 0.367 | -.21, 1.23 | 0.167 | 0.202 | 0.205 |
| Emotional response (BIPQ) | 63 | 7.78 (1.86) | 6.9 (2.32) | -0.798 | 0.259 | -1.30, -.29 | 0.002*** | -0.384 | -0.459 |
| Acceptance (ACHC) | 64 | 6.83 (6.07) | 9.29 (6.25) | 1.828 | 0.607 | .64, 3.02 | 0.003*** | 0.295 | 0.466 |

Note. ACHC = Acceptance of Chronic Health Condition Scale; BIPQ= Brief Illness Perception Questionnaire; CBRQ= Cognitive and Behavioural Responses to Symptoms Questionnaire; SEMCD= Self-Efficacy of Managing Chronic Diseases; SRM=standardised response mean; UK IBDQ= UK IBD Quality of Life Questionnaire

^$^ 12-week data presented only for those who responded (*missing completely at random* (MCAR) assumption), even though the mixed model is based on a *missing at random* (MAR) assumption.

### Appendix 8. Forest plot of adjusted treatment effect sizes for putative mediating variables

Note. ACHC = Acceptance of Chronic Health Condition Scale; BIPQ= Brief Illness Perception Questionnaire; CBRQ= Cognitive and Behavioural Responses to Symptoms Questionnaire; SEMCD= Self-Efficacy of Managing Chronic Diseases

Error bars represent 95% confidence intervals

### Appendix 9. Table comparing ethnicity between study samples (IMPARTS and COMPASS-IBD) and population sample for Lambeth, Southwark & Kent

|  | Population  n= 2,239,214 | COMPASS-IBD sample  n=65 | IMPARTS sample  n=827 |
| --- | --- | --- | --- |
| Asian | 5.7% | 10.8% | 7.8% |
| Black | 9.1% | 6.2% | 6.1% |
| Mixed | 4.0% | 4.6% | 4.2% |
| Other | 2.1% | 3.1% | 3.9% |
| White | 79.1% | 75.4% | 77.9% |

*Note*. Census data from each of the three areas was extracted and synthesised to estimate the ethnic breakdown of the catchment area.

# References

1. Kroenke K, Strine TW, Spitzer RL, Williams JB, Berry JT, Mokdad AH. The PHQ-8 as a measure of current depression in the general population. Journal of affective disorders. 2009;114(1-3):163-73.

2. Spitzer RL, Kroenke K, Williams JB, Löwe B. A brief measure for assessing generalized anxiety disorder: the GAD-7. Archives of internal medicine. 2006;166(10):1092-7.

3. Prins A, Bovin MJ, Smolenski DJ, Marx BP, Kimerling R, Jenkins-Guarnieri MA, et al. The primary care PTSD screen for DSM-5 (PC-PTSD-5): development and evaluation within a veteran primary care sample. J Gen Intern Med. 2016;31(10):1206-11.

4. Sekhon M, Cartwright M, Francis JJ. Acceptability of healthcare interventions: an overview of reviews and development of a theoretical framework. BMC health services research. 2017;17:1-13.

5. Finch T, Girling M, May C, Mair F, Murray E, Treweek S, et al. NoMAD: implementation measure based on normalization process theory. 2015.

6. Greenhalgh T, Maylor H, Shaw S, Wherton J, Papoutsi C, Betton V, et al. The NASSS-CAT tools for understanding, guiding, monitoring, and researching technology implementation projects in health and social care: protocol for an evaluation study in real-world settings. JMIR research protocols. 2020;9(5):e16861.

7. Murray E, Treweek S, Pope C, MacFarlane A, Ballini L, Dowrick C, et al. Normalisation process theory: a framework for developing, evaluating and implementing complex interventions. BMC medicine. 2010;8:1-11.

8. Powell BJ, Waltz TJ, Chinman MJ, Damschroder LJ, Smith JL, Matthieu MM, et al. A refined compilation of implementation strategies: results from the Expert Recommendations for Implementing Change (ERIC) project. Implementation science. 2015;10:1-14.

9. Kroenke K, Wu J, Yu Z, Bair MJ, Kean J, Stump T, et al. The patient health questionnaire anxiety and depression scale (PHQ-ADS): Initial validation in three clinical trials. Psychosomatic medicine. 2016;78(6):716.

10. Kroenke K, Spitzer RL, Williams JBW. The PHQ-9 - Validity of a brief depression severity measure. J Gen Intern Med. 2001;16(9):606-13.

11. Yalcin I, Bump RC. Validation of two global impression questionnaires for incontinence. Am J Obstet Gynecol. 2003;189(1):98-101.

12. Cheung W-y, Garratt AM, Russell IT, Williams JG. The UK IBDQ—a British version of the inflammatory bowel disease questionnaire: development and validation. Journal of clinical epidemiology. 2000;53(3):297-306.

13. Broadbent E, Petrie KJ, Main J, Weinman J. The brief illness perception questionnaire. Journal of psychosomatic research. 2006;60(6):631-7.

14. Picariello F, Chilcot J, Chalder T, Herdman D, Moss‐Morris R. The Cognitive and Behavioural Responses to Symptoms Questionnaire (CBRQ): Development, reliability and validity across several long‐term conditions. British Journal of Health Psychology. 2023.

15. Lorig K, Stewart A, Ritter P, Gonzalez V, Lynch J, Laurent D. Outcome measures for health education and other health care interventions: Sage; 1996.

16. Stuifbergen A, Becker H, Blozis S, Beal C. Conceptualization and development of the acceptance of chronic health conditions scale. Issues in mental health nursing. 2008;29(2):101-14.
